# Supplementary material for: Multi-level evidence of an allelic hierarchy of USH2A variants in hearing, auditory processing and speech/language outcomes
Source: Commun Biol. 2020 Apr 20;3:180. doi: 10.1038/s42003-020-0885-5 (PMC7170883; doi:10.1038/s42003-020-0885-5)
Supplement: Supplementary file 1 — Supplementary Information [file 42003_2020_885_MOESM1_ESM.pdf]

Online Supplement for *Multi-level evidence of an allelic hierarchy of USH2A variants in hearing, auditory processing and speech/language outcomes*

Supplementary Figures 2

Supplementary Figure 1: *Ush2a* ultrasonic vocalization syllable repertoire generated from MUPET 2

Supplementary Figure 2: Manhattan Plot of genomewide interaction study 3

Supplementary Tables 4

Supplementary Table 1: Language and hearing candidate genes 4

Supplementary Notes, Discussion, Methods 5

MEMBERSHIP OF WGS500 5

Supplementary Figures

Supplementary Figure 1: Ush2a ultrasonic vocalization syllable repertoire generated from MUPET

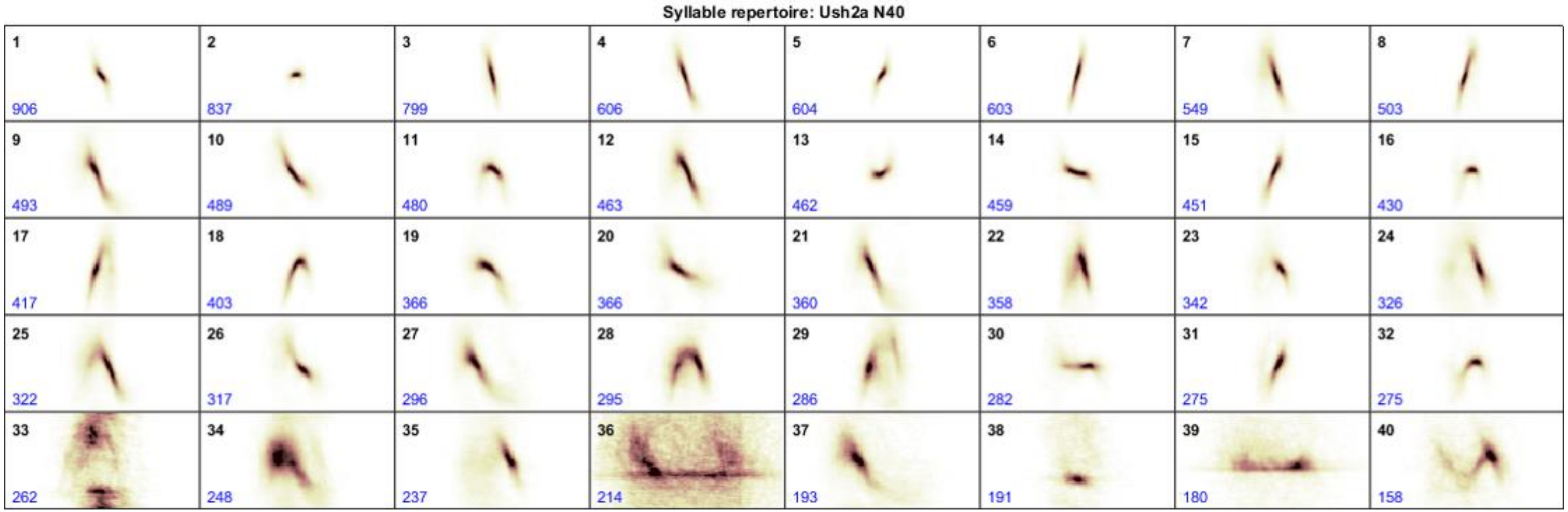

40 unique syllables were generated via MUPET USV analysis (collapsed across all subjects). Each syallable was assigned to one of eight call types [80]. Number in bottom left corner indicates number of utterances seen across all subjects.

Supplementary Figure 2: Manhattan Plot of genomewide interaction study

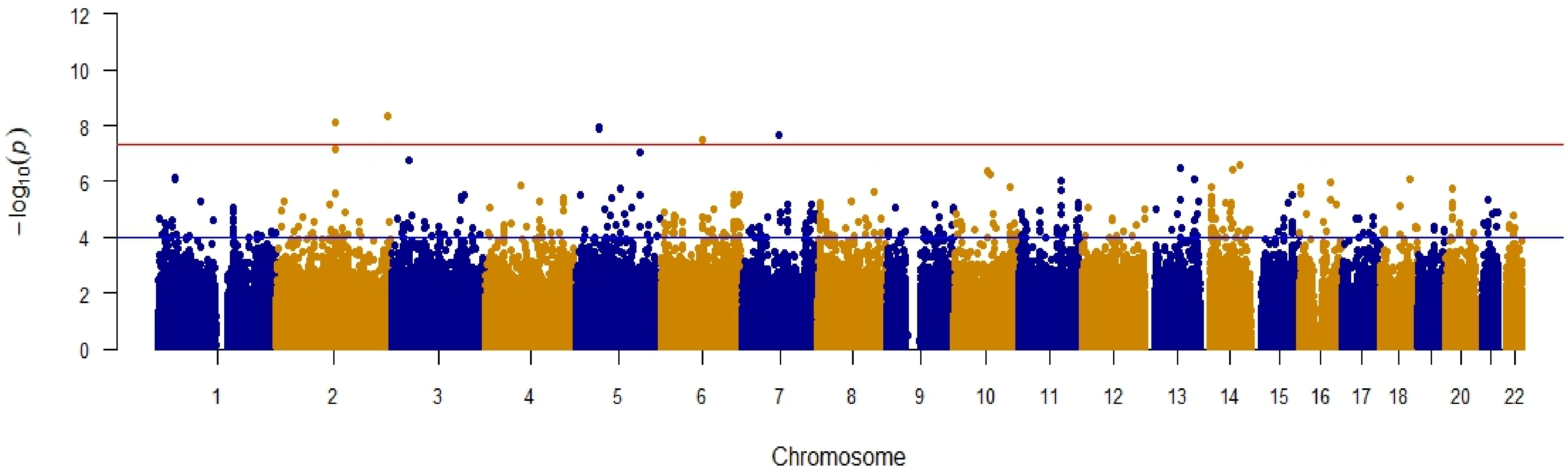

Four hundred and fifty SNPs had P-values≤10-5 and were entered into pathway analyses to identify gene pathways modulated by low frequency hearing.

Supplementary Tables

Supplementary Table 1: Language and hearing candidate genes

Hearing-related Genes						
AAK1	*COL11A1*	*EPS8L2*	*IFNLR1*	*MYO6*	*PPIP5K2*	*TJP2*
ACSL4	*COL11A2*	*ERAL1*	*IKZF5*	*MYO7A*	*PPM1A*	*TMC1*
ACTG1	*COL2A1*	*ESPN*	*IL1R2*	*NARS2*	*PRPS1*	*TMEM132E*
ACVR2A	*COL4A3*	*ESRP1*	*ILDR1*	*NDP*	*PTPRQ*	*TMEM30B*
ADCY1	*COL4A4*	*ESRRB*	*KARS*	*NEDD4L*	*RDX*	*TMIE*
AHSG	*COL4A5*	*EWSR1*	*KCNJ10*	*NFATC3*	*ROR1*	*TMPRSS3*
AIFM1	*COL4A6*	*EYA1*	*KCNQ1*	*NIN*	*RP11-166N6.3*	*TMTC4*
ANKRD11	*COL9A1*	*EYA4*	*KCNQ4*	*NISCH*	*RP11-20I23.1*	*TNC*
AP3M2	*COL9A2*	*FAM65B*	*KIAA1024L*	*NLRP3*	*S1PR2*	*TOX*
AP3S1	*COL9A3*	*FOXI1*	*KITLG*	*NM_001702*	*SEMA3E*	*TPRN*
ATP2B1	*CRYM*	*GATA2*	*KLC2*	*NM_032119*	*SEMA3F*	*TRAM2*
BAIAP2L2	*CTC-454I21.3*	*GGA1*	*KLHL18*	*NPTN*	*SERPINB6*	*TRIOBP*
BDP1	*CYB5R2*	*GIPC3*	*LARS2*	*OCM2*	*SIX1*	***TSPEAR***
BSND	*DCDC2*	*GJB2*	*LHFPL5*	*ODF3L2*	*SIX5*	*UBE2B*
CABP2	*DFNB31*	*GJB3*	*LOXHD1*	*OSBPL2*	*SLC17A8*	*UBE2G1*
CCDC50	*DFNB59*	*GJB6*	*LRTOMT*	*OTOA*	*SLC22A4*	*USH1C*
CCDC88C	*DIABLO*	*GPR152*	*MARVELD2*	*OTOF*	*SLC26A4*	*USH1G*
CCDC92	*DIAPH1*	*GPR50*	*MCM2*	*OTOG*	*SLC26A5*	***USH2A***
CD164	*DIAPH3*	*GPR98*	*MED28*	*OTOGL*	*SLC4A10*	*VTI1A*
CDC14A	*DMXL2*	*GPSM2*	*MET*	*P2RX2*	*SLC5A5*	*WBP2*
CDH23	*DNASE1*	*GRHL2*	*MIR96*	*PAX3*	*SMPX*	*WDTC1*
CEACAM16	*DUOXA2*	*GRXCR1*	***MITF***	*PCDH15*	*SNAI2*	*WFS1*
CH507-396I9.6	*EDN3*	*GRXCR2*	*MPDZ*	*PDZD7*	*SOX10*	*ZCCHC14*
CHD7	*EDNRB*	*GSDME*	*MSRB3*	*PHF6*	*SPNS2*	
CIB2	*ELMOD1*	*HARS*	*MYH1*	*PNPT1*	*SRRM4*	
CLDN14	*ELMOD3*	*HARS2*	*MYH14*	*POLR1C*	*STRC*	
CLIC5	*EMB*	*HGF*	*MYH9*	*POLR1D*	*SYNE4*	
CLPP	*EPS8*	*HOMER2*	*MYO15A*	*POU3F4*	*TCOF1*	
COCH	*EPS8L1*	*HSD17B4*	*MYO3A*	*POU4F3*	*TECTA*	

Language-related Genes	
ARID1A	*STARD9*
ATP2C2	*SYNPR*
AUTS2	*TM4SF20*
BCL11A	*TNRC6B*
CHD3	*TRIO*
CMIP	*WDR5*
CNTNAP2	*ZFHX4*
CNTNAP5	
CTNND2	
DCDC2	
DOCK4	
DYX1C1	
ERC1	
FOXP1	
FOXP2	
GRIN2A	
GRIN2B	
KAT6A	
KIAA0319	
KMT2D	
MACROD2	
MKL2	
NFXL1	
OXR1	
ROBO1	
SCN9A	
SEMA6D	
SETBP1	
SRPX2	

Hearing-related (N=197) and language-related (N=37) genes from literature. Genes highlighted in grey and bold had SNP hits with P-values <10-5 in the GWIs

Supplementary Notes, Discussion, Methods

MEMBERSHIP OF WGS500

WGS500 Consortium membership: names and affiliations:

Steering Committee: Peter Donnelly (Chair)[1], John Bell[2], David Bentley[3], Gil McVean[1], Peter Ratcliffe[1], Jenny Taylor[1,4], Andrew Wilkie[4,5]

Operations Committee: Peter Donnelly (Chair)[1], John Broxholme[1], David Buck[1], Jean-Baptiste Cazier[1], Richard Cornall[1], Lorna Gregory[1], Julian Knight[1], Gerton Lunter[1], Gil McVean[1], Jenny Taylor[1,4], Ian Tomlinson[1,4], Andrew Wilkie[4,5]

Sequencing: David Buck (Lead)[1], Christopher Allan[1], Moustafa Attar[1], Angie Green[1], Lorna Gregory[1], Sean Humphray[3], Zoya Kingsbury[3], Sarah Lamble[1], Lorne Lonie[1], Alistair Pagnamenta[1], Paolo Piazza[1], Guadelupe Polanco[1], Amy Trebes[1]

Data Analysis: Gil McVean1 (Lead), Peter Donnelly[1], Jean-Baptiste Cazier[1], John Broxholme[1], Richard Copley[1], Simon Fiddy[1], Russell Grocock[3], Edouard Hatton[1], Chris Holmes[1], Linda Hughes[1], Peter Humburg[1], Alexander Kanapin[1], Stefano Lise[1], Gerton Lunter[1], Hilary Martin[1], Lisa Murray[3], Davis McCarthy[1], Andy Rimmer[1], Natasha Sahgal[1], Ben Wright[1], Chris Yau[6]

1 The Wellcome Trust Centre for Human Genetics, Roosevelt Drive, Oxford, OX3 7BN, UK

2 Office of the Regius Professor of Medicine, Richard Doll Building, Roosevelt Drive, Oxford, OX3 7LF, UK

3 Illumina Cambridge Ltd., Chesterford Research Park, Little Chesterford, Essex, CB10 1XL, UK

4 NIHR Oxford Biomedical Research Centre, Oxford, UK

5 Weatherall Institute of Molecular Medicine, John Radcliffe Hospital, Headington, Oxford OX3 9DS, UK

6 Imperial College London, South Kensington Campus, London, SW7 2AZ, UK
