# Supplementary material for: Multi-level evidence of an allelic hierarchy of USH2A variants in hearing, auditory processing and speech/language outcomes
Source: Commun Biol. 2020 Apr 20;3:180. doi: 10.1038/s42003-020-0885-5 (PMC7170883; doi:10.1038/s42003-020-0885-5)
Supplement: Supplementary file 6 — Description of Additional Supplementary Files [file 42003_2020_885_MOESM6_ESM.pdf]

Description of Additional Supplementary Files

Supplementary Data sets (available as additional files)

- **Supplementary Data 1 - Shared variants called in discovery family**
- **Supplementary Data 2 - SNPs with P≤10-5 in genome-wide interaction study and mapped genes**
- **Supplementary Data 3 - Data for murine auditory analyses (Data underlying Figure 2)**
- **Supplementary Data 4 - Data for murine auditory analyses (Data underlying Figure 3)**
