# Supplementary material for: Multi-level evidence of an allelic hierarchy of USH2A variants in hearing, auditory processing and speech/language outcomes
Source: Commun Biol. 2020 Apr 20;3:180. doi: 10.1038/s42003-020-0885-5 (PMC7170883; doi:10.1038/s42003-020-0885-5)
Supplement: Supplementary file 7 — Reporting Summary [file 42003_2020_885_MOESM7_ESM.pdf]

nature research

Corresponding author(s): Newbury DF

Last updated by author(s): 26/02/20

Reporting Summary

Nature Research wishes to improve the reproducibility of the work that we publish. This form provides structure for consistency and transparency in reporting. For further information on Nature Research policies, see Authors & Referees and the Editorial Policy Checklist.

Statistics

For all statistical analyses, confirm that the following items are present in the figure legend, table legend, main text, or Methods section.

n/a	Confirmed	
☐	☒	The exact sample size (n) for each experimental group/condition, given as a discrete number and unit of measurement
☐	☒	A statement on whether measurements were taken from distinct samples or whether the same sample was measured repeatedly
☐	☒	The statistical test(s) used AND whether they are one- or two-sided<br>*Only common tests should be described solely by name; describe more complex techniques in the Methods section.*
☐	☒	A description of all covariates tested
☐	☒	A description of any assumptions or corrections, such as tests of normality and adjustment for multiple comparisons
☐	☒	A full description of the statistical parameters including central tendency (e.g. means) or other basic estimates (e.g. regression coefficient) AND variation (e.g. standard deviation) or associated estimates of uncertainty (e.g. confidence intervals)
☐	☒	For null hypothesis testing, the test statistic (e.g. F, t, r) with confidence intervals, effect sizes, degrees of freedom and P value noted<br>*Give P values as exact values whenever suitable.*
☒	☐	For Bayesian analysis, information on the choice of priors and Markov chain Monte Carlo settings
☐	☒	For hierarchical and complex designs, identification of the appropriate level for tests and full reporting of outcomes
☐	☒	Estimates of effect sizes (e.g. Cohen's d, Pearson's r), indicating how they were calculated

Our web collection on statistics for biologists contains articles on many of the points above.

Software and code

Policy information about availability of computer code

Data collection	Adobe Audition, DIGIcheck
Data analysis	PLINK, RVTESTS, STRING, MUPET, R (v3.4.4), Platypus, qqman, stampy, Picard, VCFtools, SNPeff (v3.2), PennCNV, QuantiSNP, SPSS (v24)

For manuscripts utilizing custom algorithms or software that are central to the research but not yet described in published literature, software must be made available to editors/reviewers. We strongly encourage code deposition in a community repository (e.g. GitHub). See the Nature Research guidelines for submitting code & software for further information.

Data

Policy information about availability of data

All manuscripts must include a data availability statement. This statement should provide the following information, where applicable:

- Accession codes, unique identifiers, or web links for publicly available datasets
- A list of figures that have associated raw data
- A description of any restrictions on data availability

A summary of variants found in the discovery family is provided in the supplementary dataset. ALSPAC and UK10K SNP and sequence data are available upon application as outlined at http://www.bristol.ac.uk/alspac/researchers/access/. The ALSPAC website additionally contains details of all the data that is available through a fully searchable data dictionary and variable search tool (http://www.bristol.ac.uk/alspac/researchers/our-data/).

Field-specific reporting

Please select the one below that is the best fit for your research. If you are not sure, read the appropriate sections before making your selection.

☒ Life sciences ☐ Behavioural & social sciences ☐ Ecological, evolutionary & environmental sciences

For a reference copy of the document with all sections, see nature.com/documents/nr-reporting-summary-flat.pdf

Life sciences study design

All studies must disclose on these points even when the disclosure is negative.

Sample size	The discovery family included twelve individuals in total. Seven members of the discovery family (five affected non-founder individuals and two unaffected Founder individuals) were genotyped on Illumina HumanOmniExpress-12v1 Beadchips (San Diego, CA, USA; ~750,000 SNPs) allowing haplotype reconstruction. This sample size was limited by the pedigree size and individual availability. Two of the most distantly related individuals were subject to whole genome sequencing .<br>Phenotypic outcomes were investigated in fourteen individuals all of whom carried heterozygous loss of function variants in the USH2A gene. This sample was limited by the frequency of these variants (estimated at 1.1%).<br>The replication cohorts included SNP data for 7141 children (3615M:3526F), providing 96% power to detect a variant that explains 0.5% of the trait variance at a Bonferroni-corrected alpha level of 7.87x10-5 and sequence data for 1681 individuals (806M:875F) providing 81% power to detect a variant that explains 1% of the trait variance at a Bonferroni-corrected alpha level of 0.0033.<br>Thirty five mice (Twelve WT male mice, 12 HT male mice, and 11 Ush2a KO male mice) underwent behavioral testing.
Data exclusions	Both replication cohorts were filtered to include only individuals with available phenotype data, of British ethnicity, born at more than 32 weeks gestation and a birth weight >1500g. Additional filters were applied for the analysis of common variation in the ALSPAC cohort. These aimed to exclude children with overt pathology that may confound language development, namely non-verbal IQ<65 and hearing loss (hearing thresholds above 40dbL).
Replication	Findings in the discovery family were replicated through the ascertainment of 14 individuals with heterozygous loss of function variants in the USH2A gene.<br>Investigation of behavioral outcomes were replicated within a mouse knockout model.<br>Additional exploration of outcomes were performed within a population cohort consisting of 7141 children (3615M:3526F) with SNP data and 1681 individuals (806M:875F) with sequence data.
Randomization	Animal behaviours were compared between wild-type, heterozygous and full knockout groups.<br>Association analyses of genetic data were quantititative and thus included all available individuals without sub-grouping.
Blinding	Behavioral testing was performed blind to subject genotype.

Reporting for specific materials, systems and methods

We require information from authors about some types of materials, experimental systems and methods used in many studies. Here, indicate whether each material, system or method listed is relevant to your study. If you are not sure if a list item applies to your research, read the appropriate section before selecting a response.

Materials & experimental systems

n/a	Involved in the study	
☒	☐	Antibodies
☒	☐	Eukaryotic cell lines
☒	☐	Palaeontology
☐	☒	Animals and other organisms
☐	☒	Human research participants
☒	☐	Clinical data

Methods

n/a	Involved in the study	
☒	☐	ChIP-seq
☒	☐	Flow cytometry
☒	☐	MRI-based neuroimaging

Animals and other organisms

Policy information about studies involving animals; ARRIVE guidelines recommended for reporting animal research

Laboratory animals	Experimental mice were re-derived on an 129S4/SvJaeJ background strain. Behavioural testing included 35 male juvenile mice
Wild animals	This study did not include wild animals
Field-collected samples	This study did not include animals collected in the field

Ethics oversight	All procedures were conducted in compliance with the Guide to the Care and Use of Laboratory Animals and was approved by the University of Connecticut's Institutional Animal Care and Use Committee (IACUC).

Note that full information on the approval of the study protocol must also be provided in the manuscript.

Human research participants

Policy information about studies involving human research participants

Population characteristics	The ALSPAC population cohort includes children born to 14541 mothers from Avon in 1991. All pregnant mothers were invited to participate in the ALSPAC study regardless of age and ethnicity. Individuals were followed throughout life and requested to complete questionnaires and testing each year. Participants are now 27-28 years old. The present study excluded individuals without genotype and relevant behavioural data. In order to homogenise genetic effects, we excluded individuals of non-British ethnicity. Behavioural data were analysed from ages 3, 7 and 8.
Recruitment	Recruitment to ALSPAC was opportunistic and aimed to recruit women as early in pregnancy as possible. ALSPAC attempted to make contact with eligible women through media information encouraging study contact and recruitment staff visiting community locations. In parallel, routine antenatal and maternity health services were used to promote the study and distribute an 'expression of interest' card. Through returning this card, women were able to request further information on the study or to decline participation. Completed cards contained sufficient detail (address and EDD) to allow ALSPAC staff to determine eligibility. Women requesting further information were sent a study information booklet followed by an initial questionnaire 1 week later. Invitation cards indicated that study consent was 'opt out', i.e. women not actively declining participation would be included in future data collection follow-up.
Ethics oversight	Ethical approval for the discovery family was provided by University of London & St George's University Hospitals. All members provided informed consent/assent of investigation. Ethical approval for ALSPAC was obtained from the ALSPAC Ethics and Law Committee and the Local Research Ethics Committees (http://www.bristol.ac.uk/alspac/researchers/research-ethics/).

Note that full information on the approval of the study protocol must also be provided in the manuscript.
